# Synthesis and Characterization of TiO_2_ Nanotubes (TiO_2_-NTs) Decorated with Platine Nanoparticles (Pt-NPs): Photocatalytic Performance for Simultaneous Removal of Microorganisms and Volatile Organic Compounds

**DOI:** 10.3390/ma14237341

**Published:** 2021-11-30

**Authors:** Lotfi Khezami, Imen Lounissi, Anouar Hajjaji, Ahlem Guesmi, Aymen Amine Assadi, Brahim Bessais

**Affiliations:** 1 Department of Chemistry, College of Sciences, Imam Mohammad Ibn Saud Islamic University (IMSIU), P.O. Box 5701, Riyadh 11432, Saudi Arabia; amalkasme@imamu.edu.sa; 2 Laboratoire de Photovoltaïque, Centre de Recherches et des Technologies de l’Energie, Technopole de Borj-Cédria, BP 95, Hammam-Lif 2050, Tunisia; lounissiimen2019@gmail.com (I.L.); physicshajjaji@gmail.com (A.H.); bessaisb@gmail.com (B.B.); 3 École Nationale Supérieure de Chimie de Rennes, Univ Rennes, CNRS, ISCR (Institut des Sciences Chimiques de Rennes) – UMR 6226, F-35000 Rennes, France

**Keywords:** Pt-NPs/TiO_2_ nanotubes, microorganism inactivation, batch reactor, indoor air treatment, kinetic modeling

## Abstract

This work reports on the effect of TiO_2_ nanotubes (TiO_2_-NTs), decorated wih platinum nanoparticles (Pt-NPs), on the removal of bacteria and volatile organic compounds (VOCs). The Pt-NPs were loaded onto the TiO_2_-NTs using the electrodeposition method at four decoration times (100, 200, 300, and 600 s). The realized Pt-NPs/TiO_2_-NTs nanocomposites were used for the degradation of cyclohexane, a highly toxic and carcinogenic VOC pollutant in the chemical industry. The achieved Pt-NPs/TiO_2_-NTs nanocomposites were characterized using X-ray diffraction (XRD), photoluminescence (PL), diffuse reflectance spectroscopy (UV–Vis), and scanning (SEM) and transmission (TEM) electron microscopy. To understand the photocatalytic and antibacterial behavior of the Pt-NPs/TiO_2_-NTs, simultaneous treatment of Escherichia coli and cyclohexane was conducted while varying the catalyst time decoration. We noticed a complete bacterial inactivation rate with 90% VOC removal within 60 min of visible light irradiation. Moreover, the Langmuir–Hinshelwood model correlated well with the experimental results of the photocatalytic treatment of indoor air.

## 1. Introduction

Today, humans spend most of their time in confined spaces (homes, transport compartments, and offices). In addition, in a constrained energy context, residential and tertiary buildings are increasingly insulated and airtight. Therefore, indoor air quality (IAQ) is becoming a significant public health concern [1]. Non-specific pollution (linked to human occupation) is characterized by a diffuse and fluctuating pollution according to activities and by a smaller pollutant concentration range (of the order of ppm or ppb) but can be dangerous for health at repetitive and lasting exposure. The main organic compounds involved are formaldehyde, cyclohexane, benzene, and toluene [2]. Moreover, the indoor air of most public spaces (hospitals, libraries, etc.) is highly contaminated according to the European Commission Classification [2,3]. Furthermore, most isolates are considered potential candidates for the establishment of sick building syndromes (SBS) and are often associated with clinical manifestations such as allergies, rhinitis, asthma, and conjunctivitis [3,4]. This SBS can be due to the presence of bacteria, viruses, and pollens. Thus, special attention must be given to controlling the keys environmental factors that lead to the growth and multiplication of microbes in public, closed indoor environments to protect the health of users and workers. This situation forces industrialists to reduce emissions by either revising processes and/or installing their effluents’ purification systems [5]. These are of a great variety, and each has its strengths and weaknesses. Indeed, some systems integrating various purification techniques (mechanical particle and molecular filters, electrostatic filters, and catalysis under UVC irradiation) were tested. The results show a diversity of performance profiles, and the overall treatment efficiencies have been unsatisfactory [3,4,5]. The photocatalytic process with visible light was found to be an indoor air remediation solution using materials, such as catalysts, with antimicrobial effects [6,7,8]. Recently, several studies have been developed around TiO_2_ tubular nanostructures thanks to their high specific surface area [9], excellent biocompatibility, and high uniformity. Nanoarchitectured titania can be fabricated using a variety of techniques including metalorganic chemical vapor deposition [10], sol-gel synthesis [11], hydrothermal [12], and anodization of titanium [13,14,15,16,17,18,19]. Among these techniques, our interest was focused on the anodization of titanium foil, which enables the achievement of self-organized TiO_2_ nanotube arrays with the easiness of geometry control (length, diameter, and wall thickness) via application of suitable anodization parameters. The fine-tuning of the dimensional features of the nanotube arrays facilitates their application in various fields, including dye-sensitized solar cells [20], biosensors [21,22], and highly efficient photocatalysis systems [23,24,25]. Despite its ability to be used in various applications TiO_2_ suffers from its restricted optical absorption in the visible range and the high recombination rate of photogenerated electron–hole pairs. Indeed, TiO_2_ is a wide band gap (3.2 eV for anatase) semiconductor that absorbs light only in the ultraviolet frequency range, representing only 4% of the solar spectrum [26], which significantly hampers its photocatalytic efficiency in the direct use of sunlight. Consequently, it is essential to develop an effective solution to improve the efficiency of charge separation and the photoactivity of TiO_2_ nanotubes by doping with metals (Ag [27,28,29,30], Cu, Au, etc.) or sensitizing with metallic nanoparticles or semiconductors (PbS [31,32] and Cu_2_O [33,34]) or decorating the surface with nanostructured noble metals (Pt [35] and Pd [36]).

This investigation aimed to prepare and characterize Pt-NPs/TiO_2_-NTs nanocomposites by depositing Pt nanoparticles onto entire TiO_2_ NTs structures using the electrodeposition method. The photocatalytic efficiency of the Pt/TiO_2_-NTs under UV–Visible light was studied to remove microorganisms and volatile organic compounds (VOCs). To our knowledge, there is no study on the catalytic activity of Pt/TiO_2_-NTs for the elimination of double pollution (i.e., microorganisms and VOC) in indoor air.

## 2. Experimental

### 2.1. Preparation of TiO_2_-NTs and Pt/TiO_2_-NTs Photoelectrodes

#### 2.1.1. Elaboration of Nanotubes by Anodic Oxidation of Ti

TiO_2_ nanotubular layers were prepared from Ti substrates in the form of square plates having a surface of 2.5 cm × 2.5 cm, a thickness of approximately 1 mm, and 99.7% of purity. Surface preparation of the Ti substrates is essential before proceeding to the oxidation and anodization of titanium. The purpose of this step is to avoid possible adhesion problems and also to make the surface active. In the first step, the titanium substrates were mechanically polished using abrasive paper with different grain sizes, ranging from 320 to 2000, to make the titanium metal surface flat and shiny. In the second step, the obtained substrates were dipped in an acetone bath, then in a methanol bath, and afterward in an ultrapure water bath (15 min for each cleaning step) under ultrasound and at room temperature. Finally, the substrates were air-dried.

The TiO_2_ nanotubes were elaborated by the electrochemical anodization method in two steps. The first anodization was carried out for 45 min (as a minimum) under a constant voltage of 60 V at a temperature of 25–27 °C in a stirred electrolytic bath containing 100 mL of ethylene glycol (EG), 1% Vol. of ammonium fluoride (NH_4_F), and 2% Vol. of ultrapure water.

We kept almost the same conditions for the second anodization step and only changed the anodization time from 45 to 120 min. Finally, the TiO_2_ nanotubular layer formed on the Ti plate was immersed in an isopropan-2-ol (C_3_H_8_O) bath under ultrasound for 2 min to enhance the fixation of the nanotubular layer, as the formed nanotubes are very fragile. After, the sample was rinsed well in hot ultrapure water to remove ions resulting from the use of isopropyl alcohol. Finally, it was dried in air.

The crystallization step of the TiO_2_ nanotubes was achieved after thermal annealing in air for 3 h at 400 °C. In these conditions, we obtained crystallized anatase TiO_2_, which is known to have the highest photocatalytic activity compared to the other two existing phases (i.e., rutile and brookite).

#### 2.1.2. Decoration of TiO_2_ NTs with Platinum Nanoparticles

The electrodeposition of Pt nanoparticles on the developed TiO_2_ NTs substrates was performed using a three-electrode electrochemical cell. The electrodeposition process of Pt-NPs on the TiO_2_ nanotube substrate (working electrode) was carried out at room temperature at a constant cathodic potential of −0.12 V (vs. Ag/AgCl) for different durations (i.e., 100, 200, and 300 s) while magnetically stirring the electrolyte bath (100 mL). The latter contained 1.5 mL of 3 mM chloroplatinic acid (as a cationic precursor) and 2.75 mL of 0.5 M sulfuric acid. Further details on the synthesis of such Pt/TiO_2_-NTs composites are illustrated elsewhere [35].

### 2.2. Batch Reactor

**Figure 1:**
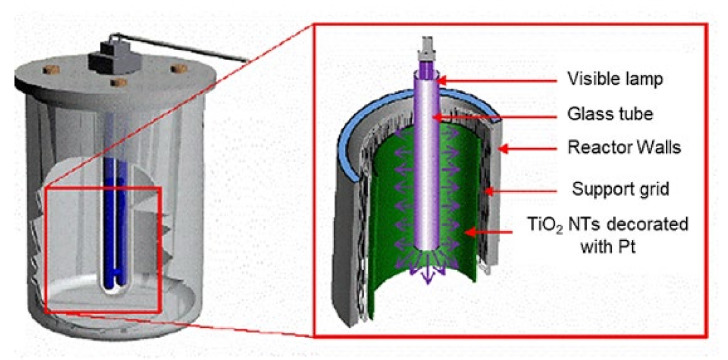
Image of the batch reactor used for the photocatalytic performance of the simultaneous removal of microorganisms and volatile organic compounds.

The photocatalytic performances of the TiO_2_-NTs and Pt-NPs/TiO_2_-NTs at different deposition times (i.e., 100, 200, and 300 s) were evaluated throughout cyclohexane degradation under visible light irradiation. The catalysts (dimensions: 1.2 cm × 2.5 cm) were placed at the bottom of the photocatalytic reactor. An ultraviolet filter was installed to block ultraviolet radiation. The initial cyclohexane concentration was set at different values of 4–20 mg/m^3^. The concentration of CYCLO was monitored by gas chromatography using a flame ionization detector (FID) (Focus GC Thermo). Nitrogen was used as a carrier gas. The temperature conditions of the oven, the injection chamber, and the detector were, respectively, 50, 150, and 200 °C. The samples were performed by injection on GC-FID with a 250 µL syringe. Before switching-on the lamp, the assembly was kept in the dark for one hour to achieve the adsorption–desorption equilibrium between the pollutant cyclohexane and the catalyst. The batch reactor is shown in Figure 1. Its height was approximately 150 mm for a volume of 500 mL. The catalytic support was deposited on the tank, and the pollutant was injected into a hermetically closed chamber with a drop and then evaporated under gentle heating. Then, a series of sampling and dilution were carried out to infiltrate the appropriate volume into the reactor according to the desired concentrations (4–20 mg/m^3^). A magnetic stirrer and a magnetic barrel made it possible to homogenize the gases.

## 3. Results

### 3.1. Characterizations of Pt-NPs/TiO_2_-NTs

Scanning electron microscopy (TESCAN VEGA3) and energy-dispersive X-ray spectroscopy (EDS) were performed to examine the nanostructured morphology and to obtain elemental analysis of the samples. The TEM and HRTEM images were obtained with an FEI Tecnai G20 microscope operating at 200 kV and equipped with a LaB6 filament. The crystalline structure and phase identification of Pt nanoparticles were determined using an X-ray diffractometer (Cu Ka radiation, λ = 1.5406 Å, PANalytical B.V., Almelo, The Netherlands). The reflectivity measurements and spectral absorption were carried out utilizing a PerkinElmer Lambda 950 UV–Visible–NIR spectrophotometer. The photoluminescence (PL) spectra were recorded with a PerkinElmer spectrophotometer equipped with a xenon lamp at an excitation wavelength λ = 340 nm.

#### 3.1.1. Scanning Electron Microscopy

**Figure 2:**
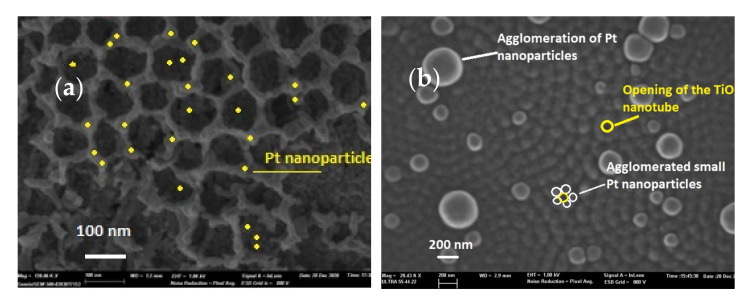
SEM surface images of TiO_2_-NTs decorated with Pt-NPs: (**a**) after 200 s and (**b**) after 300 s.

To study the morphological properties of TiO_2_-NTs decorated with Pt-NPs, we used SEM and transmission electron microscopy (TEM). Figure 2a exhibits an SEM image of TiO_2_ nanotubes decorated with Pt nanoparticles electrodeposited over 200 s. This image shows well-formed, well-immobilized, and vertically aligned nanotubular layers on the Ti substrates. The TiO_2_-NTs had an average diameter size of approximately 100 nm. It should be noted that there existed free and agglomerated Pt-NPs having a size of the order of ~10 nm on the edges and inside the TiO_2_-NTs.

Figure 2b shows a SEM image of TiO_2_ nanotubes decorated with Pt nanoparticles electrodeposited over 300 s. Visibly, the Pt-NPs agglomerated further and further as the deposition time increased. In fact, the light spots appearing at a 300 s deposition time were large Pt particles formed from an agglomeration of several NPs. This was the consequence of an increased deposition time, and it was verified by EDX analysis.

#### 3.1.2. EDX Study of the Chemical Composition of TiO_2_-NTs Decorated with Pt-NPs

**Figure 3:**
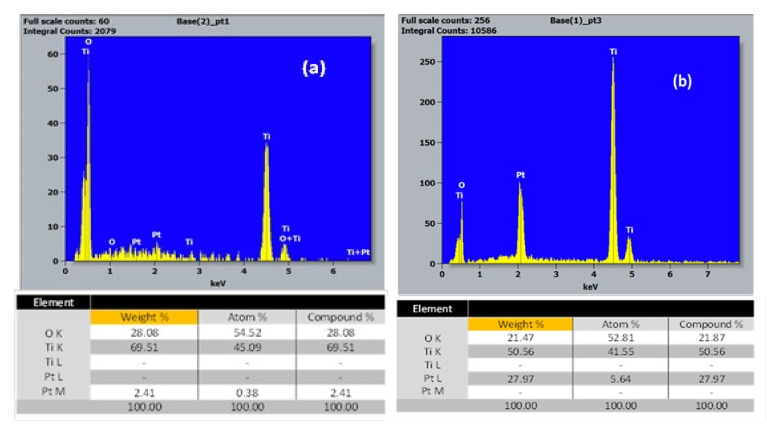
EDX spectrum of TiO_2_-NTs decorated with Pt-NPs: (**a**) 200 and (**b**) 600 s.

The chemical composition of the TiO_2_-NTs decorated with Pt-NPs was qualitatively studied by EDX using a microanalyzer coupled to the SEM. Figure 3a,b show the EDX spectrum taken on the wall of one of the TiO_2_ nanotubes for two different Pt electrodeposition times (i.e., 200 and 600 s).

These EDX spectra revealed the presence of titanium, oxygen, and platinum for both deposition times on the nanotube walls. According to the obtained results, one can notice that the Pt concentration for a 200 s deposition time was negligible compared to that for a deposition time of 600 s. This remarkable increase was due to the formation of Pt-NPs agglomerates and the beginning of the development of a film covering the TiO_2_ nanotubes.

#### 3.1.3. Transmission Electron Microscopy Characterization

**Figure 4:**
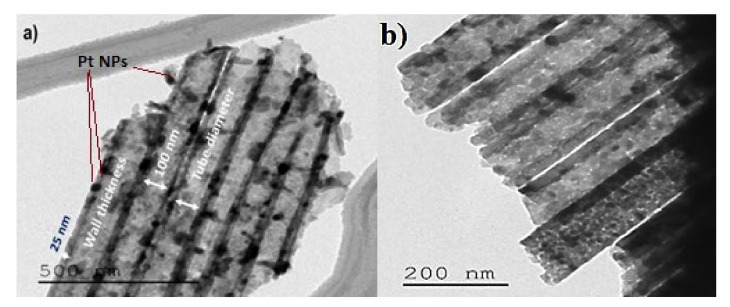
TEM images of TiO_2_-NTs decorated with Pt-NPs over (**a**) 200 and (**b**) 600 s.

TEM images (Figure 4) show that the adopted methodology promoted the formation and growth of highly ordered and structured TiO_2_ nanotubes, having an average inner diameter of approximately 100 nm and a thickness of nanotube walls of approximately 25 nm.

#### 3.1.4. X-ray Diffraction Characterization

**Figure 5:**
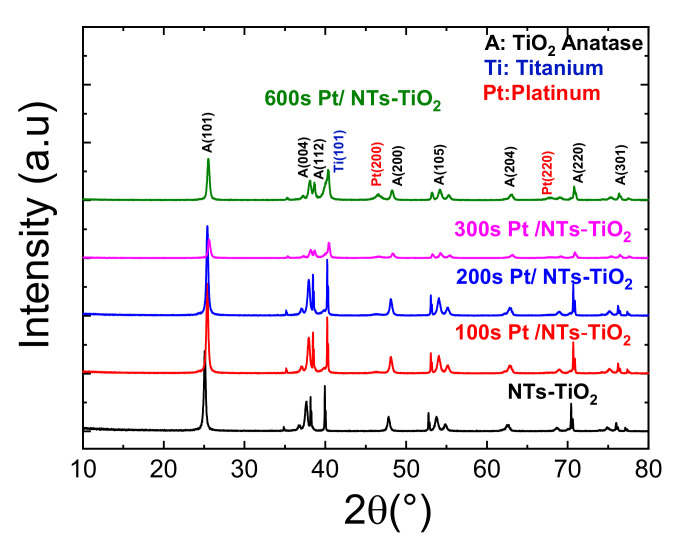
X-ray diffraction patterns of pure TiO_2_-NTs decorated with Pt-NPs.

The X-ray diffraction analysis permitted the determination of the crystalline phase, the elemental lattice parameters, and approximately the crystallite size. Figure 5 depicts the XRD patterns of pure TiO_2_-NTs annealed at 400 °C and decorated with Pt-NPs at different electrodeposition times. It can be perceived that all samples crystallized in the anatase phase. Additional peaks characterizing the presence of Pt-NPs are clearly observable, beginning from a deposition time of 300 s; below this time, the amount of Pt incorporated was insufficient to be detected, probably due to the sensitivity threshold of the XRD [37,38].

The diffraction peaks obtained at 2θ = 25.35°, 36.96°, 37.95°, 38.54°, 48.12°, 53.98°, 55.13°, 62.88°, 68.88°, 70.60°, 75.18°, and 76, 32° corresponded to the (101), (103), (004), (112), (200), (105), (211), (204), (116), (220), (215), and (301) orientation planes of the anatase phase of TiO_2_, respectively. The peaks at 2θ = 35.14°, 40.33°, 53.15°, and 77.59° were due to the diffraction from the titanium substrate. The Pt-related peaks corresponded to the (200) and (220) crystallographic orientation of the Pt face-centered cubic structure that well agreed with the JCPDS N° 70–2075 [39].

The XRD peak intensities of the TiO_2_-NTs decorated with Pt-NPs decreased as the electrodeposition time increased. This phenomenon was due to the aggregation of the Pt-NPs as the Pt electrodeposition time increased.

The main XRD peak related to the (101) orientation slightly shifted towards higher angles as the Pt-NPs’ deposition time increased. The FWHM of the TiO_2_ (101) peak remained quasi constant as the Pt electrodeposition time increased. Therefore, by only looking at the Bragg relation, this shift would be due to the lattice parameter contraction. The overall stress field sustained by the Pt-NP agglomeration is believed to have caused the observed lattice parameter contraction.

#### 3.1.5. Reflectivity Properties Investigations

**Figure 6:**
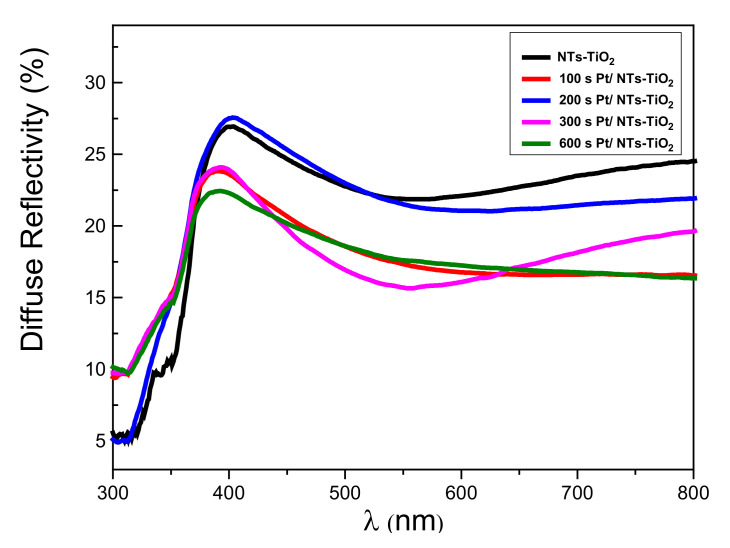
The diffuse reflectance spectrum of TiO_2_-NTs decorated with Pt-NPs.

TiO_2_-NTs decorated with Pt-NPs at different electrodeposition times were characterized by UV–Vis spectroscopy in the diffuse reflection mode (Figure 6).

**Figure 7:**
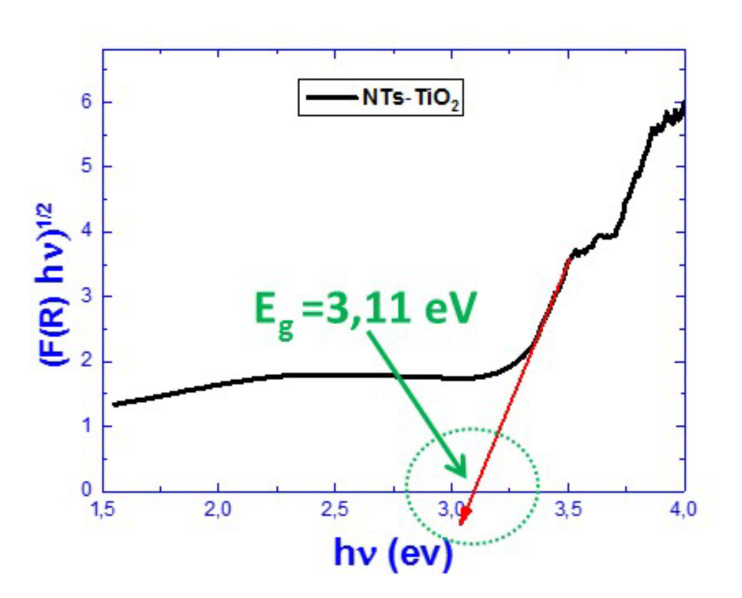
Kubelka–Munk transform of the diffuse reflectivity spectrum of pure TiO_2_-NTs.

The exploitation of the curves of the diffuse reflectivity allowed us to verify the influence of the Pt-NPs on the band gap of the TiO_2_-NTs (Figure 7). For this purpose, we used the Kubelka–Munk method [40], which connects the absorption coefficient α to the diffuse reflection coefficient *R*, according to the following equation [40]:

$$\alpha =\frac{{\left(1-R\right)}^{2}\;}{2R}$$

One may notice (in Table 1) a general decrease in the TiO_2_-NTs’ band gap energy values as the deposition time of Pt increased. This behavior broadens the absorption band of the TiO_2_-NTs for an efficient photocatalytic experiment in the visible range.

The decrease in the optical band-gap energy of TiO_2_ could be due to the increase in Pt-NP–induced defects that introduce energy levels in the band gap of TiO_2_.

#### 3.1.6. Photoluminescence Properties Investigations

**Figure 8:**
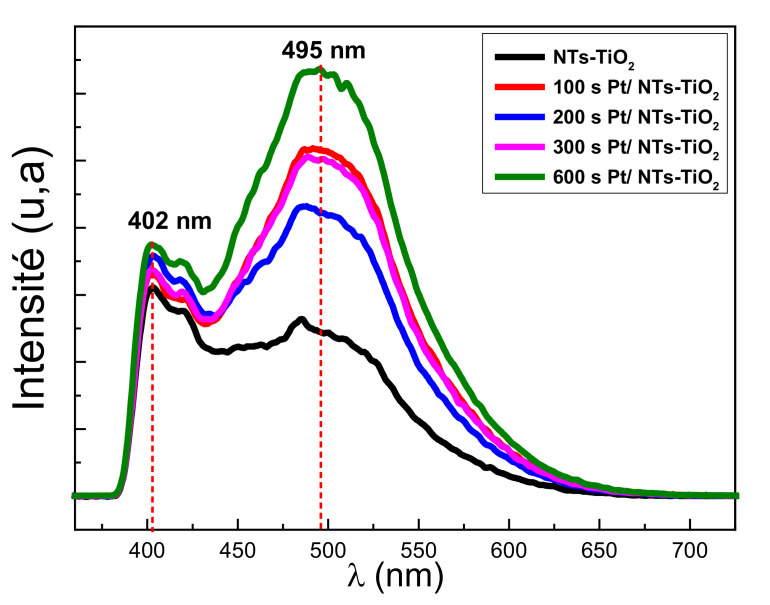
Photoluminescence spectra of TiO_2_-NTs decorated with Pt-NPs at different electrodeposition times.

Photoluminescence (PL) spectroscopy can provide information regarding the charge transfer and recombination of photogenerated charge carriers in TiO_2_-NTs [41]. Figure 8 shows the PL spectra of TiO_2_-NTs decorated with Pt-NPs at different times.

The peak localized at 402 nm corresponded to the electronic transition between the conduction and the valence bands of TiO_2_. The replica located around 495 nm represents a group of photoluminescence peaks attributed to oxygen vacancies present in TiO_2_ [42]. The decoration with Pt-NPs did not qualitatively change the form of the PL emission. The lowest PL intensity value amongst the Pt-NPs-decorated samples corresponded to those decorated with Pt-NPs for 200 s. For this electrodeposition, the Pt-NPs had a relatively small size (Figure 4). This Pt-NPs small size would have a work function higher than that of TiO_2_. In this case, the metal/SC (TiO_2_) contact was of a Schottky-type; thus, the electrons would be transferred from TiO_2_-NTs to Pt-NPs. This type of transfer minimizes the recombination rate of electron–hole pairs in TiO_2_ and would cause a low recombination rate and a low PL intensity [43].

The highest PL intensity value corresponded to TiO_2_-NTs decorated with Pt-NPs for 600 s, apparently due to the agglomeration of Pt-NPs. This agglomeration provides a work function lower than that of TiO_2_. In this situation, the metal/SC contact (TiO_2_) was ohmic, so there would be a transfer of electrons from the Pt-NPs to the TiO_2_-NTs, inducing an increase in the electron–hole recombination rate which, in turn, would increase the PL intensity (Figure 8).

### 3.2. Catalyst Application for Pollution Treatment

#### 3.2.1. Effect of Decoration Time

**Figure 9:**
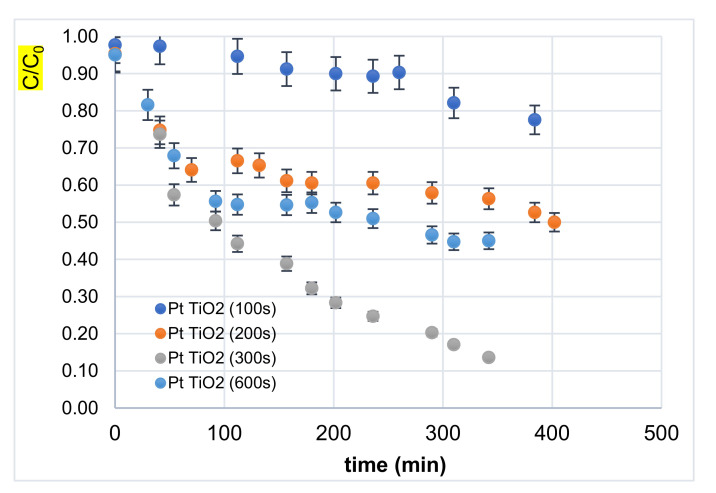
Normalized concentration (C/C_0_) of CYCLO versus Pt-NP decoration time.

Figure 9 shows the variation of the normalized concentration (C/C_0_) as a function of the irradiation time at different Pt-NP decoration times. It appears that the catalyst with 300 s decoration exhibited better performance. This behavior could be due to the greater availability of photocatalytic sites [8,44] and probably to the plasmonic effects in Pt-NPs. In fact, light irradiation would have generated plasmons in the Pt-NPs that led to a transfer of hot electrons in the TiO_2_ conduction band and, thus, a separation of positive and negative plasmons. In such a situation, hot electrons (transferred to TiO_2_) would degrade the polluting agent via a chemical reduction, while Pt positive charges would serve to catalyze the oxidation process.

On the other hand, the results manifest that degradation follows a pseudo-first-order kinetics model, where degradation is proportional to the input concentration at low values. The number of molecules effectively participating in the reaction did not increase proportionally to the input concentration, thus leading to a decrease in the degradation efficiency, which may result from the limited adsorption capacity of the active sites at the catalyst surface. In fact, the results show that at a low input concentration, degradation followed pseudo-first order kinetics, where degradation was proportional to the input concentration. This finding can be explained by the fact that all the active sites are not occupied. Moreover, an increase in the concentration generates a higher surface coverage, implying a better rate of degradation [8,44].

The efficient photocatalytic performance (Figure 9) is mainly explained by plasmon-based charge separation which, in turn, leads to better photoactivity in the visible light domain.

#### 3.2.2. Effect of Inlet Concentration

**Figure 10:**
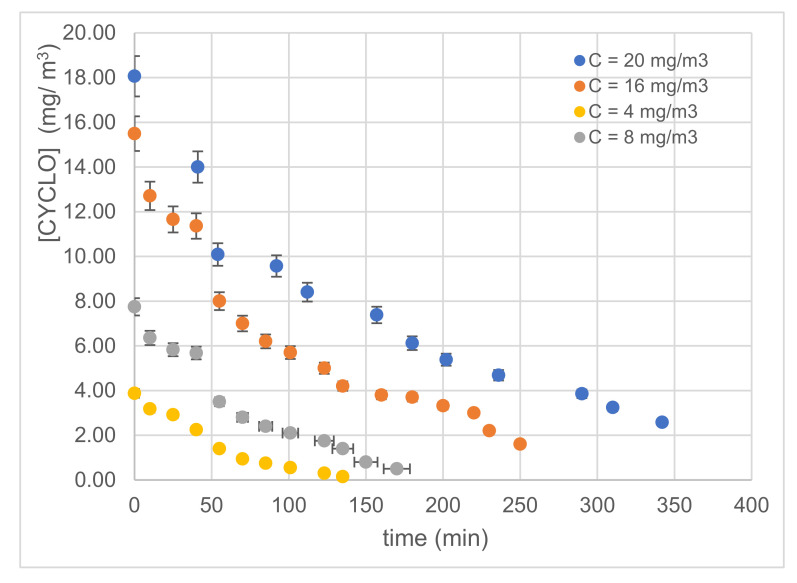
Variations in the concentration of CYCLO using Pt-NPs/TiO_2_-NTs-300 s at different inlet concentrations.

Figure 10 displays the variations in cyclohexane concentration as a function of irradiation time at different input concentrations using the most efficient catalyst (i.e., 300 s). The total pollutant removal decreased as the cyclohexane concentration at the inlet increased (Figure 10); this behavior is owed to the greater availability of photocatalytic sites at a low initial concentration [7,8,33,44,45].

In order to describe the photocatalytic performance of Pt-NPs/TiO_2_-NTs-300 s, we used the Langmuir–Hinshelwood model (L–H) equation [7,8]:

$$r_{0}=-\frac{d\left[\mathrm{CYCLO}\right]}{\mathrm{dt}}=k_{c}\frac{K{\left[\mathrm{CYCLO}\right]}_{0}}{1+K{\left[\mathrm{CYCLO}\right]}_{0}}$$

where *r*_0_ (mmol/g_cat_ m^3^ s) is the initial photocatalytic degradation rate, [*CYCLO*]_0_ is the initial cyclohexane concentration (mmol/m^3^), *K* is the adsorption constant (m^3^/mg), and *k_c_* is the kinetic constant (mg.m^−3^ min) at maximum coverage of the experimental conditions.

The plot of 1/*r*_0_ versus 1/[*CYCLO*]_0_ allows for the determination of the *k_c_* and *K* values. The linearized (L–H) equation is [7,8]:

$$\frac{1}{r_{0}}=\frac{1}{k_{c}K}\times \frac{1}{{\left[\mathrm{CYCLO}\right]}_{0}}+\frac{1}{k_{C}}$$

The kinetic and the adsorption constant of the L–H values are summarized in Table 2. The results show that the Pt-modified TiO_2_-NTs led to a faster VOC removal compared to other types of catalysts, such as TiO_2_-impregnated polyester (PES) [7] and Cu_2_O-modified TiO_2_-NTs [33], and had a kinetic constant of 0.85 mg.m^−3^min^−1^. Table 1 gives the kinetic and adsorption constants referring to the (L–H) model.

#### 3.2.3. Simultaneous Oxidation of Cyclohexane and Bacteria

**Figure 11:**
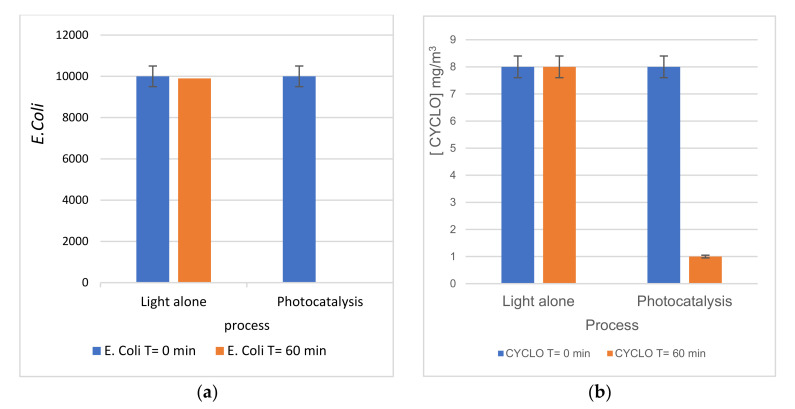
(**a**) E. coli inactivation rate in the presence of CYCLO using the Pt-NPs/TiO_2_-NTs-300 s catalyst. The initial concentration of E. coli was a ~1.1 × 10^3^ CFU/mL) (**b**) variation of the CYCLO concentration in the presence of *E. Coli*.

To investigate the antibacterial aspect in the simultaneous removal of E. coli and cyclohexane, several experiments were conducted with Pt-NPs/TiO_2_-NTs-300 s catalyst with an initial bacterial concentration of ~1.86 × 10^3^ (CFU/mL). Figure 11 shows the antibacterial activity of Pt-NPs/TiO_2_-NTs-300 s with and without cyclohexane under visible light. There was almost no notable effect of photolysis and VOC pollutants on the studied bacteria cells’ inactivation (Figure 11). Moreover, the results show that the Pt-NPs/TiO_2_-NTs-300 s sample catalyst had a complete bacterial inactivation rate with VOCs and 99.59% within 60 min of visible light irradiation.

Based on these results, Pt presents a high bacterial inactivation capability. Undoubtedly, visible light could improve the stimulation of electron transfer between Pt and bacterial cells, which leads to the formation of reactive oxygen species (ROS), resulting in microorganism inactivation [7,45,46,47]. In their previous investigations, Abidi et al. [33] indicated that the contact between some metals, such as copper species and microorganisms’ cells, led to the extraction of electrons from cells, which allows for protein denaturation. In addition, TiO_2_-NTs present an excellent antibacterial activity under UV irradiation due to the photogeneration of electron/hole pairs, resulting in ROS generation (OH^•^, O_2_^−•^, etc.) [7,33,46].

## 4. Conclusions

The formation of Pt-NPs on TiO_2_-NTs was carried out using the electrodeposition technique. The catalytic characterization results prove that the presence of Pt-NPs increased the absorption of visible light (plasmonic effect) up to approximately 500 nm. The photocatalytic degradation of 20 mg/m^3^ of cyclohexane in a batch reactor made it possible to select the catalyst with an optimized electroplating time (Pt-NPs/TiO_2_-NTs-300 s). The L–H model was used to model the degradation kinetics.

The constants obtained in our present work were compared with other metallic NPs such as copper. The optimized catalyst was also used for the simultaneous treatment of cyclohexane and microorganisms. This attractive photocatalytic efficiency was due to the surface plasmon resonance in Pt-NPs and, consequently, to a high visible light absorption. Indeed, this catalytic configuration presents an interesting bacterial inactivation. In fact, visible irradiation enhances the stimulation of electron transfer between Pt and microorganism cells through the engendering of ROS, pointing to bacterial disinfection.

## Figures and Tables

**Table 1:** Optical band gap energy of TiO_2_ NTs versus Pt deposition time.

Sample	TiO_2_-NTs Pure	100 s Pt/TiO_2_ NTs	200 s Pt/TiO_2_ NTs	300 s Pt/TiO_2_ NTs	600 s Pt/TiO_2_ NTs
**Band Gap (eV)**	3.11	2.85	2.91	2.91	2.85

**Table 2:** L–H constants (kc and K) on Cu_2_O-NPs/TiO_2_-NTs-250 s catalyst.

k_c_: Kinetic Constant of L–H (mg.m^−3^. min^−1^)	K: Adsorption Constant of L–H (m^3^. mg^−1^)
0.064	5.27

## Data Availability

Not applicable.
